# Superhydrophobic Paper-Based Microfluidic Field-Effect Transistor Biosensor Functionalized with Semiconducting Single-Walled Carbon Nanotube and DNAzyme for Hypocalcemia Diagnosis

**DOI:** 10.3390/ijms23147799

**Published:** 2022-07-15

**Authors:** Hui Wang, Ruipeng Chen, Fan Zhang, Zhixue Yu, Yue Wang, Zhonglin Tang, Liang Yang, Xiangfang Tang, Benhai Xiong

**Affiliations:** 1 State Key Laboratory of Animal Nutrition, Institute of Animal Science, Chinese Academy of Agricultural Sciences, Beijing 100193, China; wanghui10@caas.cn (H.W.); chen_ruipeng@yeah.net (R.C.); zhangfan19@139.com (F.Z.); 82101205310@caas.cn (Z.Y.); wangyue9313@163.com (Y.W.); tangzhonglin@caas.cn (Z.T.); yangliang@caas.cn (L.Y.); 2 State Key Laboratory of Animal Nutrition, College of Animal Science and Technology, China Agricultural University, Beijing 100193, China

**Keywords:** octadecyltrichlorosilane, microfluidic, cellulose paper, electrochemical biosensor, chemiresistive, calcium ion

## Abstract

Hypocalcemia is caused by a sharp decline in blood calcium concentration after dairy cow calving, which can lead to various diseases or even death. It is necessary to develop an inexpensive, easy-to-operate, reliable sensor to diagnose hypocalcemia. The cellulose-paper-based microfluidic field-effect biosensor is promising for point-of-care, but it has poor mechanical strength and a short service life after exposure to an aqueous solution. Octadecyltrichlorosilane (OTS), as a popular organosilane derivative, can improve the hydrophobicity of cellulose paper to overcome the shortage of cellulose paper. In this work, OTS was used to produce the superhydrophobic cellulose paper that enhances the mechanical strength and short service life of MFB, and a microfluidic field-effect biosensor (MFB) with semiconducting single-walled carbon nanotubes (SWNTs) and DNAzyme was then developed for the Ca^2+^ determination. Pyrene carboxylic acid (PCA) attached to SWNTs through a non-covalent π-π stacking interaction provided a carboxyl group that can bond with an amino group of DNAzyme. Two DNAzymes with different sensitivities were designed by changing the sequence length and cleavage site, which were functionalized with SPFET/SWNTs-PCA to form Dual-MFB, decreasing the interference of impurities in cow blood. After optimizing the detecting parameters, Dual-MFB could determine the Ca^2+^ concentration in the range of 25 μM to 5 mM, with a detection limit of 10.7 μM. The proposed Dual-MFB was applied to measure Ca^2+^ concentration in cow blood, which provided a new method to diagnose hypocalcemia after dairy cow calving.

## 1. Introduction

Hypocalcemia, caused by a sharp decline in blood calcium concentration after dairy cow calving, is a nutritional and metabolic disease for dairy cows [1,2]. More than 50% of dairy cows suffer from milk fever, which seriously affects the production performance and economic benefit. There are three primary forms of calcium in the blood of dairy cows: free calcium or ionic calcium (50~60%), protein-bound calcium (30%), and other bound calcium. If the Ca^2+^ concentration in blood ranges from 800 μM to 1050 μM, the dairy cow suffers from subclinical hypocalcemia, which might induce retained placenta, endometritis, displacement of the true stomach, mastitis, ketosis, etc. The cut-off blood Ca^2+^ value for clinical hypocalcemia is lower than 800 μM, causing muscle weakness, lying on the ground, loss of appetite, limb paralysis, and even coma or death. Therefore, it is necessary to develop an inexpensive, easy-to-operate, reliable sensor for detecting the blood calcium concentration, contributing to diagnosing and treating postpartum hypocalcemia after dairy cow calving.

The microfluidic field-effect biosensor (MFB) is attractive for point-of-care applications in public health, food safety, and environmental monitoring [3]. It combines the advantages of the microfluidic chip and electrochemical field-effect biosensor, including sample pretreatment, a high sensitivity, short detection time, and amenability to revolutionary device miniaturization [4,5]. Conventional MFBs fabricated using silicon, glass, or polymer requires expensive instruments and complex processes, limiting its low-cost production and use. Therefore, finding new material for the MFB’s substrate is urgent. 

Cellulose paper (CP) is a non-toxic, environmentally friendly, and mass-produced renewable material [6]. It is made of low-cost cellulose fibers through hydrogen bonds [7], has biocompatibility and piezoelectricity, is biodegradable, and enables passive liquid transport [8,9], making it a promising MFB substrate. However, the abundant hydroxyl (-OH) on CP’s surface results in the spreading of the biomaterials, which makes it difficult to modify the electrochemical biosensor [10]. In addition, a CP-based MFB in a high humidity environment results in its swelling, with poor mechanical strength [11] and a short service life [12]. Thus, it is necessary to improve the hydrophobic performance of CP.

Currently, the appropriate union of a rough surface structure and surface chemical composition is the general strategy to construct hydrophobic paper [13,14], and can form the nano-/microscale roughness on the surface and then modify the surface using low surface energy materials [15]. Due to the limitations of ideal nanostructure templates or simple fabrication procedures, it is still challenging to find hydrophobic coatings that are easy to manufacture, stable, and low-cost in practical applications. Octadecyltrichlorosilane (OTS) [16], a popular organosilane derivative, is a commonly used reagent for hydrophobic surface modification. Zhang et al. [17] report a hydrophobic solution based on a single-step, stoichiometrically controlled reaction of long-chain organosilanes with water that can create micro- to nanoscale hierarchical siloxane aggregates dispersible in industrial solvents. Different materials that obtained excellent superhydrophobicity were simply dipped into or sprayed with the coating mixture, in which, the water contact angle can reach >170°. In addition, ultraviolet light can turn oxygen in the air into ozone and atomic oxygen and oxygen, which can decompose OTS into carbon dioxide and water vapour [18,19,20]. Therefore, OTS coupled with ultraviolet light to regulate the hydrophilicity and hydrophobicity of CP, is very suitable for preparing MFB. In addition, due to ultra-sensitivity detection, mass-production capability, and low-cost manufacturing, semiconducting single-walled carbon nanotube field-effect transistor (SWNT-FET)-based biosensors [21] stand out for detecting trace substances. Thirdly, DNAzymes have been used extensively in biosensing due to their short detection time, high sensitivity, and excellent accuracy [22,23]. Zhou et al. [24] reported that a DNAzyme named EtNa-C5T was sensitive to the Ca^2+^. An inexpensive and straightforward approach used OTS to decorate cellulose paper to improve the hydrophobicity, and was then applied as the MFB’s substrate. 

In this work, we developed a CP-based MFB that was used to diagnose hypocalcemia in cow blood. Semiconducting single-walled carbon nanotubes (SWNTs) and pyrene carboxylic acid (PCA) were mixed in organic solvents as biological signal amplification material and immobilized on the channel between the source and drain directly. Two DNAzymes with different sensitivities were designed by changing the sequence length and cleavage site and were functionalized with SPFET/SWNTs-PCA to form Dual-MFB, decreasing the interference of impurities in cow blood.

## 2. Results and Discussion

### 2.1. Mechanism

The mechanism of superhydrophobic cellulose paper: the reaction of OTS with H_2_O produces trimethoxyoctadecylsilane and hydrochloric acid, resulting in the generation of much of the hydroxyl group. The condensation reaction of hydroxyl groups in trimethoxyoctadecylsilane can create different micro- to nanoscale hierarchical siloxane aggregates that are dispersible, and hexane can then be added to form a superhydrophobic solution. Based on a single-step, stoichiometrically controlled reaction of long-chain organosilanes with water, the superhydrophobic solution can create micro- to nanoscale hierarchical siloxane aggregates that are dispersible in industrial solvents. The hydroxyl groups on siloxane aggregates can react with the hydroxyl group of cellulose fiber to form chemical bonds, and the C18 long alkyl chains reduce the reaction rate when exposed to water or moisture [17,25]. When SP was treated by lithography, ultraviolet light could turn oxygen in the air into ozone and atomic oxygen and oxygen, which can decompose OTS into carbon dioxide and water vapor. The C18 long alkyl chain of OTS molecules was decomposed to form polar oxygen-containing moieties such as ketones, aldehydes, and carboxylates in Graphic abstract [26,27].

The mechanism of SPFET/SWNTs-PCA/DNAzyme for Ca^2+^ detection [28]: as shown in Figure S1, the DNAzyme molecule displays a negative charge characteristic because the phosphate groups in water can dissociate hydrogen ions; semiconducting SWNTs mainly rely on the directional movement of internal carriers (holes and electrons), especially holes; DNAzyme is immobilized on the SWNTs’ surface, which will decrease the number of internal carriers that show a high electrical resistance; when DNAzyme is exposed to Ca^2+^, EtNa-C5T can specifically bind to Ca^2+^, cleaving the ‘rA’ site located at the complementary substrate, which will affect the internal carriers (holes and electrons) of SWNTs.

### 2.2. Characterization

**Figure 1:**
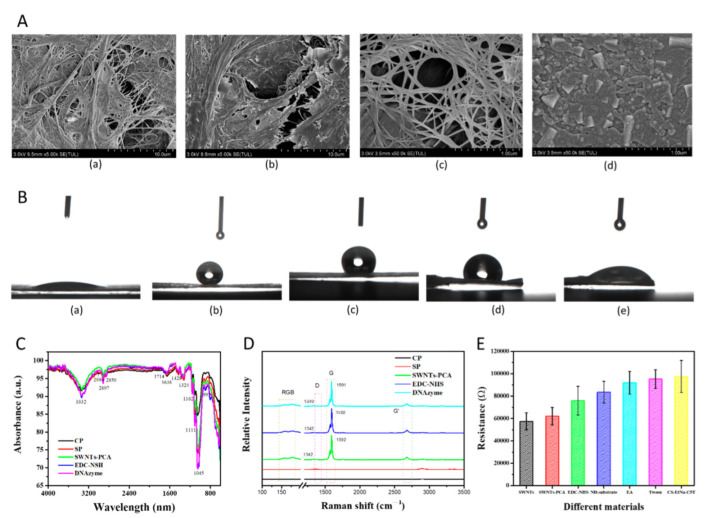
(**A**) SEM images of (a) CP, (b) SP,(c) SP/SWNTs-PCA, and (d) SP/silver paste; (**B**) WCA, (**C**) FTIR, and (**D**) Raman of CP functionalized with OTS, SWNTs-PCA, EDC-NHS, and DNAzyme; (**E**) resistances of SPFET modified with different materials (PCA, EDC-NHS, ND-substrate, EA, Tween 20, and CD-EtNa-C5T) at −0.1 V; each data point was an average of measurements from 3 independent biosensors.

The surface morphology was characterized by scanning electron microscopy (SEM) at an acceleration voltage of 3 kV, as shown in Figure 1A. All samples were sprayed with gold nanoparticles to improve the surface conductivity. CP had a rough surface, where the cellulose fibers could be clearly observed. After superhydrophobic solution treatment, the SP surface presented many flake-like structures but became smooth. This demonstrated that OTS reacted with water to create the micro- to nanoscale hierarchical siloxane aggregates that were dispersible [17], which were successfully grafted on the cellulose fiber. When SP was decorated with SWNTs-PCA, there was a high-density network structure on the surface [29]. Once the silver paste was screen-printed on SP, it formed a dense and thick film but existed as a massive structure.

Figure 1B shows water contact angles (WCA) of CP functionalized with different materials. As for CP, the water droplet was penetrated that WCA was almost 0°. After superhydrophobic treatment, the WCA reached 151°, with a high hydrophobicity, indicating that OTS was immobilized on the CP surface. When SWNTs-PCA, EDC-NHS, and DNAzyme were immobilized on the SP, the WCAs decreased gradually to 127°, 98°, and 78° because the modified materials provided the hydrophilic groups with an affinity to water [30]. 

FTIR measurements in Figure 1C show that, for CP, absorption peaks were located at 3332 cm^−1^ (-OH stretching vibration), 2897 cm^−1^ (C-H stretching vibration), 1638 cm^−1^ (vibration of water molecules), 1428 cm^−1^ and 1375 cm^−1^ (C-H deformation), 1329 cm^−1^ (CH_2_ wagging), 1162 cm^−1^ (C-O-C asymmetric vibration), 1111 cm^−1^ (glucose ring stretch), 1058 cm^−1^, and 1032 cm^−1^ (C-O stretching vibration), which corresponded to cellulose [31,32]. After OTS modification, three characteristic peaks located at 2980 cm^−1^, 2850 cm^−1^, and 1462 cm^−1^ were obviously enhanced, which corresponded to the methyl group (-CH_3_), methylene group (-CH_2_), and Si-C bond, indicating that the OTS was successfully grafted to the cellulose fiber. When SWNTs-PCA without no glucose ring and C-O-C are modified on the SP, there are changes in the intensities of the peaks at 1111 cm^−1^ and 1162 cm^−1^. The C=O characteristic peak at 1714 cm^−1^ and the intensity peaks of a C-H peak at 1162 cm^−1^ and 1111 cm^−1^ and a C-O peak at 1032 cm^−1^ provided evidence of the surface functionalization with EDC-NHS and DNAzyme.

Raman spectra of CP and SP existed in several bands in Figure 1D and Figure S2. Among them, the 1095 cm^−1^ band was assigned to the C-O-C asymmetric stretching modes along the cellulose backbone, 1119 cm^−1^ and 1127 cm^−1^ medium bands were assigned to C-C-O asymmetric stretch modes of the sugar molecules, the 2907 cm^−1^ band was assigned to C-H stretching vibrations, and the spectral regions beyond 3000 cm^−1^ were associated with O-H stretching vibrations [33]. Almost all absorption bands of SP were higher than CP, especially at 1342 cm^−1^, 1592 cm^−1^, and 2500 cm^−1^, ascribing to the generated trimethoxyoctadecylsilane. The absorption bands of CP and SP were located at the same positions because of the same chemical composition and chemical bond. Raman spectra of SPFET/SWNTs-PCA had four strong absorption bands located at 175 cm^−1^, 1342 cm^−1^, 1592 cm^−1^, and 1670 cm^−1^, which presented the radial breathing modes (RBM) region and the tangential modes (G-band) [34]. When SP functionalized SWNTs-PCA, EDC-NHS, and DNAzyme successively, the ratios of D-band to G-band in Table S1 were 0.0325, 0.0286, and 0.0263, respectively. In addition, the D and G peaks of SP/SWNT-PCA/DNAzyme shifted 2 cm^−1^ and 1 cm^−1^ in the negative direction after DNAzyme modification. The above results proved that EDC-NHS and DNAzyme had been modified with SWNTs-PCA [35,36]. 

Electrical conductivity was investigated using a linear voltage in the range from −0.2 V to +0.2 V. In Figure S3A, the slopes of *I_DS_-V_DS_* continued to decrease after SPFET/SWNTs were functionalized with different materials. For the same modification, the resistances calculated through Ohm’s law were almost equal at different voltages in Figure S3B, except for 0 V, which might be ascribed to the low-performance electrochemical workstation. In Figure 1E, the resistance of HPFET/SWNTs was 57 ± 6.2 kΩ, indicating excellent repeatability using the screen-printed method. The resistance of HPFET/SWNTs-PCA slightly increased to 61.7 ± 6.9 kΩ, ascribing to the pyrene groups of PCA bond with SWNTs through π-π stacking [37]. When EDC-NHS, EA, and Tween20 were attached to the SWNTs’ surface, the resistances were 76.8 ± 12.2 kΩ, 92.3 ± 9.2 kΩ, and 95.5 ± 7.9 kΩ, where the hole density in p-SWNTs was occupied by the negative charge of these materials [38]. For ND-substrate and CS-EtNa-C5T functionalization, the number of holes in p-SWNTs decreased remarkably due to the strong electronegativity, resulting in an increase in resistance [39,40].

### 2.3. Optimization

In order to obtain an excellent analytical performance, some parameters of SPFET/SWNTs-PCA/DNAzyme were optimized as described below.

**Figure 2:**
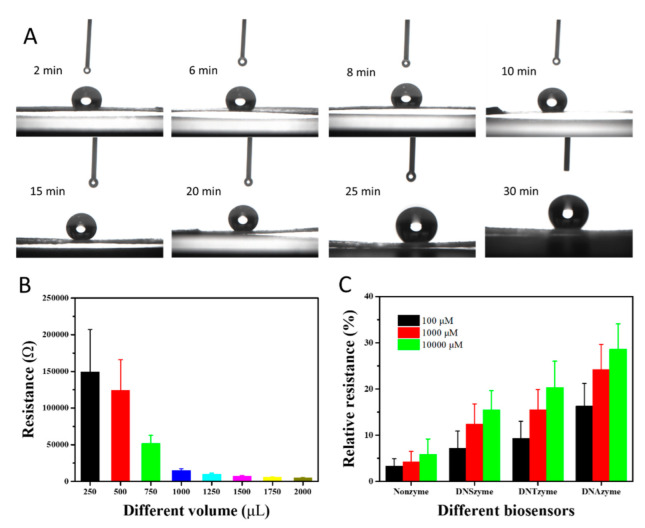
(**A**) CP-functionalized superhydrophobic solution with incubation time in the range of 2 min to 30 min.; (**B**) resistance change in SPFET/SWNTs-PCA with different volume of semiconducting ink at *V_DS_* = −0.1 V, *V_G_* = 0 V; (**C**) relative resistances changing with the structure and length of DNAzyme for three different Ca^2+^ concentrations. Each data point is an average of measurements from 3 independent biosensors.

The sufficient hydroxyl groups on CP’s surface can react with the micro- to nanoscale trimethoxyoctadecylsilane to form a superhydrophobic film. As the concentration of the superhydrophobic solution is certain, the hydrophobic property is only controlled by the immersion time. Different CPs were immersed into the superhydrophobic solution directly. At various intervals, the CPs were taken out, rinsed with hexanol, and dried under a nitrogen atmosphere. Each CP was placed on a horizontal table, deionized water was dropped on its surface, the droplet state was recorded in real-time, and then the WCA value was measured under static conditions, as shown in Figure 2A. The SP’s hydrophobicity changed significantly with the increase in immersion time. When CP was immersed for 15 min, as shown in Figure S4, the deionized water on its surface formed an approximately sphere-like droplet, where WCA reached up to approximately 151.2°. CP with superhydrophobicity can maintain mechanical strength and enhance the service life in a water environment. After that, WCA was not significantly increased and was inversely correlated with the immersion time. Therefore, the optimal immersion time was 15 min.

The resistance of SPFET/SWNT-PCA can be tuned by the volume of the SWNTs-PCA solution, which further affects the sensitivity and stability. Different volumes of semiconducting ink were dropped on the different SPFETs, and then the current–voltage curve was measured (*I_DS_-V_DS_*). The slop of *I_DS_-V_DS_* was proportional to the volume of semiconducting ink, as shown in Figure S5. The average resistance of SPFET/SWNT-PCA decreased from 150 KΩ to 3.0 KΩ, with the volume increasing from 250 to 2000 μL, as shown in Figure 2B. SPFET/SWNT-PCA prepared with a low volume will have a considerable variation in the resistance value, resulting in a low stability. On the contrary, the resistance of SPFET/SWNTs-PCA prepared with a high volume formed a thick layer, reducing the effective specific surface area, affecting the sensitivity, and increasing the energy consumption. Thus, 750 μL of the SWNTs-PCA solution was chosen to prepare the SPFET/SWNT-PCA, with an average resistance of 52.6 ± 6.7 kΩ. 

The sensitivity of MFB is strongly correlated with the number of base pairs and RNA cleavage sites of DNAzyme. Four substrates (NO-substrate, NS-substrate, NT-substrate, and ND-substrate) were designed and synthesized, as shown in Table 1, which were immobilized on SPFET/SWNTs-PCA and functionalized with CS-EtNa-C5T or CD-EtNa-C5T to form SPFET/SWNTs-PCA/Nonzyme, SPFET/SWNTs-PCA/DNSzyme, SPFET/SWNTs-PCA/DNTzyme, and SPFET/SWNTs-PCA/DNAzyme. The prepared MFBs were used to determine different Ca^2+^ concentrations (pH 7.4). In Figure 2C, the relative resistances of SPFET/SWNTs-PCA/Nonzyme for different Ca^2+^ concentrations had no significant change, and the relative resistance of SPFET/SWNTs-PCA/DNTzyme was higher than SPFET/SWNTs-PCA/DNSzyme, indicating that ‘rA’ is the cleavage site. Compared to SPFET/SWNTs-PCA/DNTzyme, the relative resistance of SPFET/SWNTs-PCA/DNAzyme increased by approximately 70% with high sensitivity. The main reason is that the electronegativity of DNAzyme was enhanced with the number of base pairs, which further affected the carrier density in SWNTs. 

Figure S6A shows the *I_DS_-V_DS_* of SPFET/SWNTs-PCA/DNAzyme before and after exposure to different Ca^2+^ concentrations. The current increased with the voltage in the range of −0.2 V to 0.2 V, which exhibited a nonlinear relationship. The reason might be ascribed to the semiconducting property of SWNTs, affected by alkaline buffer solution approximately equated to a negative base voltage. In addition, the current might appear at a peak because of the reduction reaction of silver oxide when the voltage was higher than 0 V, indicating that the silver paste was not sealed in the presence of organic silica gel. The resistance at each voltage was calculated using Ohm’s law, as shown in Figure S6B. When the voltage is lower than −0.02 V, the resistance values were approximate equality, as well as the relative resistance, as shown in Figure S6C,D. To avoid the influence, −0.1 V was chosen to simplify the detection process in the following experiment. 

The cleavage efficiency of DNAzyme is associated with the Ca^2+^ concentration and incubation time. Three different Ca^2+^ concentrations (100, 1000, and 10,000 μM) were measured by SPFET/SWNTs-PCA/DNAzyme with varying incubation times in the range of 0 to 11 min. As shown in Figure S7, the relative resistance of SPFET/SWNTs-PCA/DNAzyme was proportional to the Ca^2+^ concentrations and incubation time. For the same Ca^2+^ concentration, the relative resistance increased with incubation time, but the growth rate decreased significantly when the incubation time was higher than 7 min. For this reason, 7 min was chosen as the optimal incubation time for the experiment.

### 2.4. Selectivity

Selectivity is a critical factor for electrochemical biosensors. The cow blood serum contained many metal ions, such as Na^+^, K^+^, Mg^2+^, Zn^2+^, Cu^2+^, Fe^2+^, Fe^3+^, and Cr^3+^, which might interfere with the relative resistance of SPFET/SWNTs-PCA/DNAzyme. To evaluate the selectivity, different SPFET/SWNTs-PCA/DNAzymes were used to measure 10 mM Ca^2+^ or other metal ions under the optimized conditions. The detection results are shown in Figure S8. The relative resistance of SPFET/SWNTs-PCA/DNAzyme was approximately 28.6 for 10 mM Ca^2+^. In comparison, the relative resistances for Mg^2+^ and Zn^2+^ were much higher than those for the rest of the metal ions. Compared to the blank buffer solution, the four metal ions solutions had slightly higher relative resistances, indicating that most metal ions did not interfere with the SPFET/SWNTs-PCA/DNAzyme.

### 2.5. Linear Relationship

**Figure 3:**
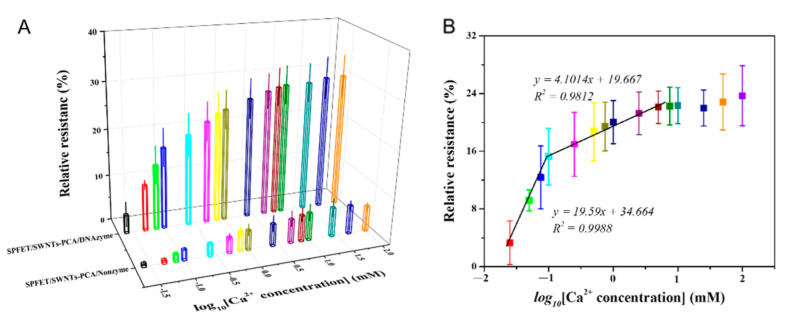
(**A**) Relative resistance of SPFET/SWNTs-PCA/DNAzyme and SPFET/SWNTs-PCA/Nonzyme for different Ca^2+^ concentrations in the range of 25 μM to 100 mM; (**B**) the linear relationship of the relative resistance and the logarithm of the Ca^2+^ concentration.

SP can effectively avoid pollution from the cow blood, but SWNTs might be interfered with by impurities in the serum. As discussed in Figure S8, minor interference from the other metal ions will affect the detection accuracy of SPFET/SWNTs-PCA/DNAzyme. Dual-MFB integrated with two MFBs on one substrate was proposed to improve the detection accuracy in the serum, which modified DNAzyme with and without ‘rA’ (SPFET/SWNTs-PCA/DNAzyme and SPFET/SWNTs-PCA/Nonzyme). Under the optimized conditions, different Ca^2+^ concentrations at pH = 7.4 were measured using SPFET/SWNTs-PCA/DNAzyme and SPFET/SWNTs-PCA/Nonzyme. As shown in Figure 3A, it was clear that the relative resistances of the two biosensors were positively correlated with Ca^2+^ concentrations at each voltage. However, the values were quite different, indicating that SPFET/SWNTs-PCA/DNAzyme still existed with weak interferences. The difference in the relative resistance of SPFET/SWNTs-PCA/DNAzyme and SPFET/SWNTs-PCA/Nonzyme can represent the actual Ca^2+^ concentration, and the linear relationships are shown in Figure S9. Based on analysis and calculation, the difference in relative resistance revealed an excellent relationship with the logarithm of Ca^2+^ concentration in two linear intervals in Figure 3B. For Dual-MFB, the regression equations were $y_{1}=22.132x+39.265$ in the low concentration range from 0.025 mM to 0.1 mM and $y_{2}=5.4265x+23.577$ in a high concentration range from 0.1 mM to 5.0 mM, with the R-squared value of 0.9939 and 0.9782, respectively. The detection limit was 10.7 μM (N/S = 3), which was significantly lower than the Ca^2+^ concentration in the serum of dairy cows (500 μM). Compared to other sensors for Ca^2+^ determination, as shown in Table S2, the proposed Dual-MFB not only had a similar performance but could also be mass-produced at a low cost. 

### 2.6. Real Sample Analysis

Practicality is most important for the electrochemical device. Blood samples were collected from the vein of the cow’s tail using a syringe, which was located in southeastern Beijing. Dual-MFB (SPFET/SWNTs-PCA/DNAzyme and SPFET/SWNTs-PCA/Nonzyme) was employed to measure the blood Ca^2+^ in dairy cows, which was compared to atomic absorption spectrometry (AAS). The blood sample was filtered by the hydrophilic channel of Dual-MFB, which can remove the hemoglobin and macromolecular proteins. The detected results are shown in Table 2. It was clear that the Ca^2+^ concentrations of samples (No. 1, No. 2, and No. 3) were lower than 1050 μM, indicating that subclinical hypocalcemia occurred in these dairy cows. Even though the detection accuracy of Dual-MFB was lower than AAS, the average recoveries ranged from 93.12 to 109.16%, and the *p*-value of the *t*-test was 0.8232 > 0.05, indicating that the proposed Dual-MFB could be used to determine Ca^2+^ concentrations with a high accuracy.

## 3. Methods and Materials

### 3.1. Chemicals and Materials

Semiconducting single-walled carbon nanotubes (SWNTs, 90%) were obtained from NanoIntegris Inc. (Menlo Park, CA, USA). Pyrene carboxylic acid (PCA, 97%), 1-ethyl-3-(3-dimethylaminopropyl)-carbodiimide hydrochloride (EDC), and N-hydroxysulfosuccinimide (NHS) were purchased from Sigma Aldrich (Beijing, China). Tween 20 was purchased from Bio-Rad (Beijing, China). Cellulose paper (Whatman No.4) was purchased from Shanghai Jinpan Biotechnology Co., Ltd. (Shanghai, China). Spraying conductive silver adhesive and organic silica gel were purchased from Shenzhen Jingzhe Technology Co., Ltd. (Shenzhen, China). Octadecyltrichlorosilane (OTS, purity > 95%) was bought from Sinopharm Chemical Reagent Co., Ltd. (Beijing, China). N-hexane (purity > 95%) was purchased from Chongqing Chuandong Chemical Group Co., Ltd. (Chongqing, China). Sodium monohydrogen phosphate and sodium dihydrogen phosphate were offered by Shanghai Yuanye Biotechnology Co., Ltd. (Shanghai, China). N, N-Dimethylformamide (DMF), acetone, ethanolamine (EA, 99%), methanol, 3-Tris(hydroxymethyl)aminomethane (Tris), hydrochloric acid (HCl), heparin lithium, sodium hydrogen phosphate, and sodium dihydrogen phosphate were obtained from the Shanghai Mackin Biochemical Technology Co., Ltd. (Shanghai, China). Potassium chloride, sodium chloride, zinc chloride, copper chloride, iron chloride, magnesium chloride, calcium chloride, and lead chloride were acquired from Sinopharm Chemical Reagent Beijing Co., Ltd. (Beijing, China). The reagents were of analytical grade without further purification, and ultrapure water from a Millipore Milli-Q system was used throughout the experiment. 

DNA oligonucleotides in this experiment were ordered from Shanghai Sangon Biotechogies Co., Ltd. (Shanghai, China). Four DNAzymes with different sequence lengths and ‘rA’ sites were designed, and are listed in Table 1. The first four substrates were dissolved in 100 μM phosphate buffer solution with pH 6.2, and the rest were dissolved in 100 μM Tris-HCl solution with pH 7.4, of which, the concentrations were measured using a UV spectrometer at 260 nm.

### 3.2. Apparatus

Surface morphology was investigated using a Hitachi Scanning Electron Microscope SU3500 (Tokyo, Japan). Water contact angle (WCA) was recorded using an OCA 50AF (Filderstadt, German). Fourier transform infrared spectrometer (FTIR) was conducted using an InfraRed Bruker Tensor-37 (Karlsruhe, Germany). Raman spectra were measured through the Renishaw InVia Raman microscope with an imaging microscope (532 nm diode and Ar ion lasers). CHI 760E electrochemical workstation (Shanghai, China) was applied to record the current-voltage (I-V) at 25 °C, where the voltage ranged from −0.2 V to +0.2 V (step +0.01 V). The source was connected to the working electrode, the drain was connected to the counter electrode, and the reference electrode was attached to CHI 760E electrochemical workstation.

### 3.3. Preparation of Superhydrophobic Cellulose Paper

**Figure 4:**
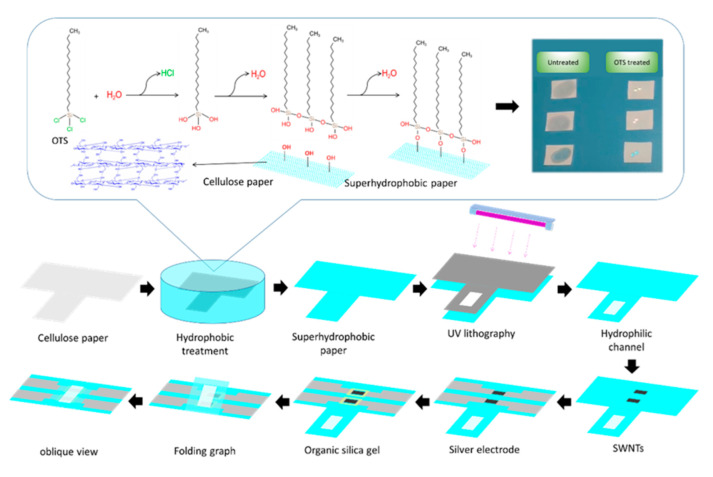
Preparing process of Dual-MFB structure using superhydrophobic cellulose paper, UV lithography, and screen-printing technology.

Figure S10 shows the preparation process of superhydrophobic solution. Briefly, water was added to pure OTS with the volume ratio (1:10) in a centrifuge tube. The tube was capped and immediately put on a vortex mixer at 4000 rpm for 20 s, then treated by an ultrasonic cleaner for 10 s (uncapped) and another round of vortex mixing for 10 s (capped). After that, the suspended emulsion was transferred to a big glass bottle with a cap immediately and then kept away from light for two hours with a cap on but not airtight. Before use, the treated solution and pure hexane with the volume ratio (1:20) were mixed by sonication in an ultrasonic cleaner to form the superhydrophobic solution. As shown in Figure 4, cellulose paper (CP) was cut into a specific size and immersed in the superhydrophobic solution prepared above for 10 min. The treated paper was rinsed with hexane several times to remove the residue and dried in an N_2_ atmosphere. The superhydrophobic effect of SP was shown in Videos S1–S3. A hydrophilic channel for blood filtration was made by decomposing superhydrophobic materials using 185 nm ultraviolet light for 10 min. 

Semiconducting ink of SWNTs-PCA was synthesized as the following protocol. Briefly, 0.1 mg SWNTs powder was dispersed in 15 mL DMF by ultrasonic treatment for 2 h. A total of 0.2 mg PCA was added into 5 mL DMF and completely dissolved by vortex oscillation treatment. The two dispersed solutions were well mixed with stirring for 12 h to achieve complete-stacking interaction. Before each use, the mixing solution needed to be treated in an ultrasonic cleaner for 30 min. Screen printing masks retained two channels (4 mm long and 2 mm wide) that were overlapped on the superhydrophobic cellulose paper. The channel was placed at the center of a vacuum filter funnel channel under a vacuum force of 2.0 MPa. Each channel was evenly coated with 750 μL SWNTs-PCA ink through a syringe. The vacuum forced the SWNTs-PCA ink onto the SP. The residues of PCA were rinsed with DMF and ethanol serval times, which were further annealed in an oven at 50 ℃. After that, the silver paste was screen-printed on each end of the sensing channel. The terminals were subsequently dried in an oven at 100 °C for 20 min. Finally, the junction between SWNTs-PCA and silver paste was blocked using organic silica gel that was hardened in the air at room temperature. The overall structural diagram is shown in Figure S11.

### 3.4. Modification of Biomaterial

**Figure 5:**
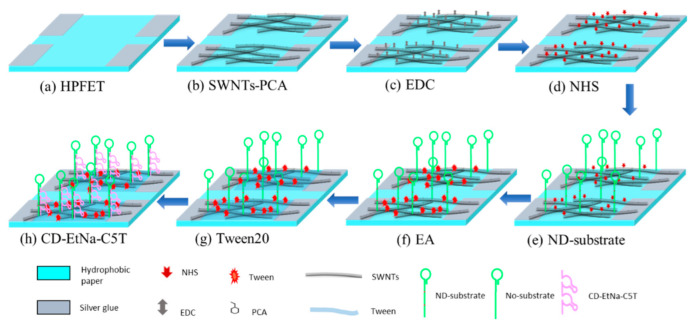
SPFET functionalized with different chemical and biological materials successively (SWNTs-PCA, EDC, NHS, ND/NO-substrate, EA, Tween20, and CD-EtNa-C5T).

Before biomaterial modification, SPFET/SWNTs-PCA was rinsed with DMF and ethanol to remove organic and inorganic residues. As shown in Figure 5, the carboxylic groups on SPFET/SWNTs-PCA were activated by phosphate buffer (50 mM, pH 6.2) containing 4 mM EDC and 8 mM NHS for 30 min and then flushed with adequate phosphate buffer solution to remove the residuals. The activation mechanism was shown in supporting Equations (S1)–(S3).

The two different amine-labeled DNA of ND-substrate and NO-substrate (100 μM, pH 6.2) were covered with the activated SPFET/SWNTs-PCA overnight at 4 °C, respectively. After that, SPFET/SWNTs-PCA/ND-substrate and SPFET/SWNTs-PCA/NO-substrate were functionalized with EA (0.1 mM, pH7.4) to block excess ester groups of PBASE and Tween 20 (0.1%, pH 7.4) to prevent nonspecific binding to SWNTs to prevent the adsorption of organic and inorganic impurities. The treated SPFET/SWNTs-PCA/ND-substrate and SPFET/SWNTs-PCA/NO-substrate were hybridized with CD-EtNa-C5T (100 μM, pH 7.4) to form a DNAzyme duplex (SPFET/SWNTs-PCA/DNAzyme and SPFET/SWNTs-PCA/Nonzyme) for 2 h at room temperature.

### 3.5. Sensing Protocol

To measure Ca^2+^ concentration, the sensing protocol was performed in the following steps: the blood sample was covered on the hydrophilic channel of Dual-MFB for 7 min at room temperature. Then, the currents of SPFET/SWNTs-PCA/Nonzyme and SPFET/SWNTs-PCA/DNAzyme were measured at −0.1 V. The resistance was calculated using R=U/I.

The relative resistance was calculated using the following equation:

$$Relative\;resistance=\left(R_{0}-R\right)/R_{0}\times 100\%\;$$

where $\;R_{0}$ was the resistance value of SPFET/SWNTs-PCA/Nonzyme or SPFET/SWNTs-PCA/DNAzyme before exposure to cow blood, and R was the resistance of SPFET/SWNTs-PCA/DNAzyme after exposure to cow blood.

## 4. Conclusions

Collectively, an inexpensive and straightforward approach using OTS was proposed to produce a superhydrophobic solution, and was then applied to decorate CP to fabricate SP. This work provided a simple and effective method to fabricate superhydrophobic cellulose paper as MFB’s substrate, where a hydrophilic channel was etched using 185 nm UV lithography as filter film. The DNAzyme molecule displays a negative charge characteristic because the phosphate groups in water can dissociate hydrogen ions. DNAzyme is immobilized on the SWNTs’ surface, decreasing the number of internal carriers that change the conductivity. SWNTs and DNAzyme were used to construct a microfluidic field-effect biosensor. The sensitivity of the MFB was obviously enhanced by changing the structure and length of the DNAzyme. The linear range was from 25 μM to 5 mM, and the detection limit was 10.7 μM. SPFET/SWNTs-PCA/DNAzyme combined with SPFET/SWNTs-PCA/Nonzyme can decrease the interference from impurities in cow blood, where the relative error was lower than 10%. An excellent detection performance with mass production and low cost make Dual-MFB suited for hypocalcemia diagnoses in the dairy cow.

## Figures and Tables

**Table 1:** DNA oligonucleotides in this study.

Name	Sequence and Modifications (from 3′-Terminus)
NS-substrate	NH_2_-(CH_2_)_6_-GCGGTAGAAGG/rA/TATCACTGAGCACTGGGATAAGCGGTAGA
NT-substrate	NH_2_-(CH_2_)_6_-GCGGTAGAAGG/rA/TATCACTGAGCACTGGG/rA/TAAGCGGTA GA
ND-substrate	NH_2_-(CH_2_)_6_-GCGGTAGAAGG/rA/TATCACTGAGCACTGGG/rA/TAAGCGGTA GAACTCACAATGTATAATGCGCGCATTATACATTGTGAGT
NO-substrate	NH_2_-(CH_2_)_6_-GCGGTAGAAGGATATCACTGAGCACTGGGATAAGCGGTAGAA CTCACAATGTATAATGCGCGCATTATACATTGTGAGT
CS-EtNa-C5T	TCTACCGCTTATCCCAGTGCTCAGTGATTGTTGGAATGGCTCATGCCACACTCTTTTCTACCGC
CD-EtNa-C5T	TCTACCGCTTTGTTGGAATGGCTCATGCCACACTCTTCAGTGCTCAGTGATTGTTGGAATGGCTCATGCCACACTCTTTTCTACCGC

**Table 2:** Ca^2+^ concentrations in the actual samples determined by Dual-MFB and AAS.

Sample	Add Ca^2+^ Concentration (μM)	Dual-MFB (μM)	AAS (μM)	Recovery (%)
1	-	821 ± 73	879	93.40
	500	1420 ± 108	-	102.97
2	-	992 ± 89	934	106.21
	500	1543 ± 142	-	107.60
3	-	812 ± 89	874	93.12
	500	1327 ± 131	-	96.58
4	-	1072 ± 93	982	109.16
	500	1401 ± 173	-	94.53

## Data Availability

Not applicable.

## Supplementary Materials

The following supporting information can be downloaded at: https://www.mdpi.com/article/10.3390/ijms23147799/s1. References [41,42,43,44,45,46,47,48,49,50,51] are cited in the Supplementary Materials.
