# Supplementary material for: Superhydrophobic Paper-Based Microfluidic Field-Effect Transistor Biosensor Functionalized with Semiconducting Single-Walled Carbon Nanotube and DNAzyme for Hypocalcemia Diagnosis
Source: Int J Mol Sci. 2022 Jul 15;23(14):7799. doi: 10.3390/ijms23147799 (PMC9318675; doi:10.3390/ijms23147799)
Supplement: Supplementary file 1 [file ijms-23-07799-s001.zip › ijms-1785367-supplementary.pdf]

Superhydrophobic paper-based microfluidic field-effect transistor biosensor functionalized with semiconducting single-walled carbon nanotube and DNAzyme for hypocalcemia diagnosis

Hui Wang [1], Ruipeng Chen [1], Fan Zhang [1,2], Zhixue Yu [1], Yue Wang [1], Zhonglin Tang [1], Liang Yang [1], Xiangfang Tang [1*] and Benhai Xiong [1*]

1 State Key Laboratory of Animal Nutrition, Institute of Animal Science, Chinese Academy of Agricultural Sciences, Beijing 100193, China; wanghui10@caas.cn (H.W.); chen_ruipeng@yeah.net (R.C.); zhangfan19@139.com (F.Z.); 82101205310@caas.cn (Z.Y.); wangyue9313@163.com (Y.W.); tangzhonglin@caas.cn (Z.T.); yangliang@caas.cn (L.Y.)

2 State Key Laboratory of Animal Nutrition, College of Animal Science and Technology, China Agricultural University, Beijing 100193, China

* Correspondence: tangxiangfang@caas.cn (X.T.); xiongbenhai@caas.cn (B.X.)

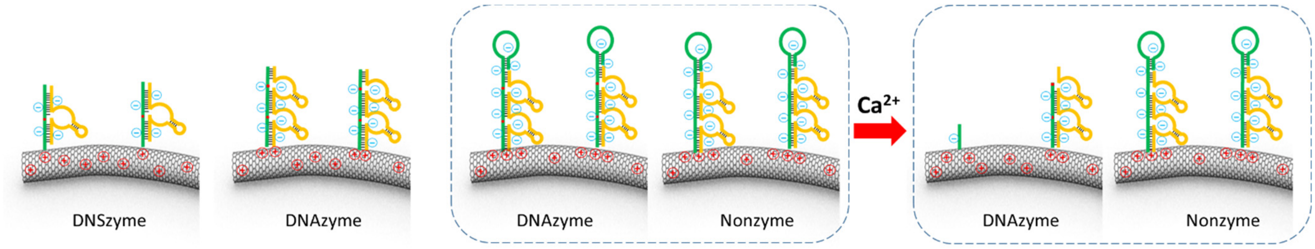

Figure S1. Mechanism of electrochemical field-effect biosensor for Ca^{2+} detection.

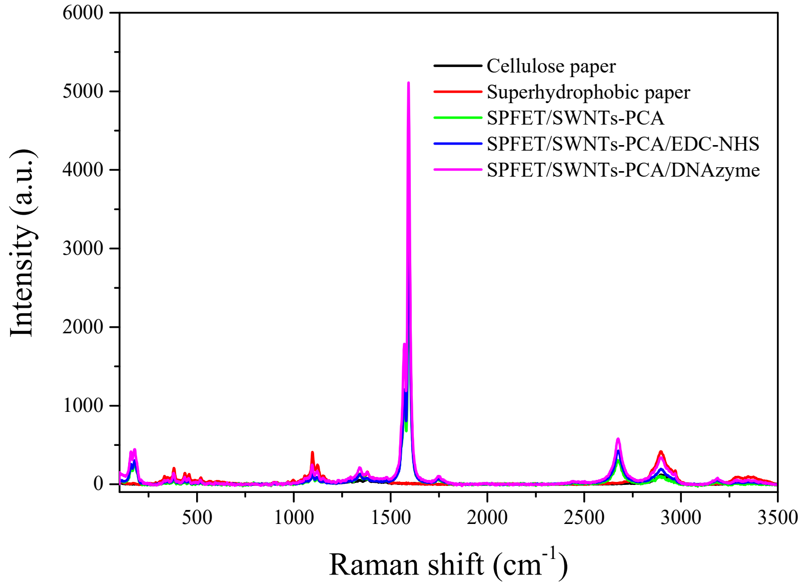

Figure S2. Raman spectra of cellulose paper before and after functionalized different materials: (a)Cellulose paper; (b)Superhydrophobic paper; (c)SPFET/SWNTs-PCA; (d)SPFET/SWNT-PCA/EDC-NHS and (e) SPFET/SWNT-PCA/DNAzyme.

Table S1. The ratios of different bands to G-band

	SWNT-PCA	EDC-NHS	DNAzyme
I_{RBM}/I_G	0.0984	0.0805	0.0814
I_D/I_G	0.0325	0.0286	0.0263
$I_{G'}/I_G$	0.109621	0.114043	0.105141

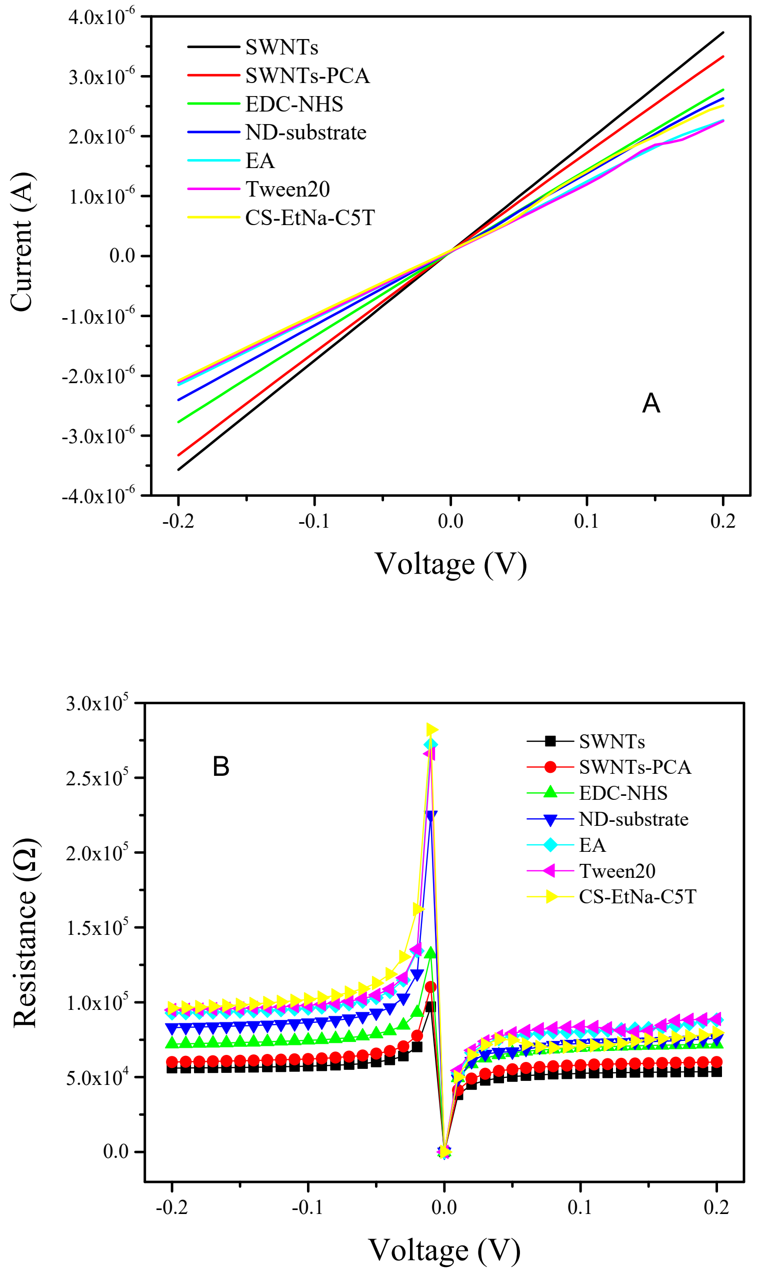

Figure S3. (A) I_{DS}–V_{DS} and (B)Resistances of HPFET modified with different materials (SWNTs, PCA, EDC-NHS, ND-substrate, EA, Tween 20 and CS-EtNa-C5T) at different voltages.

Optimization

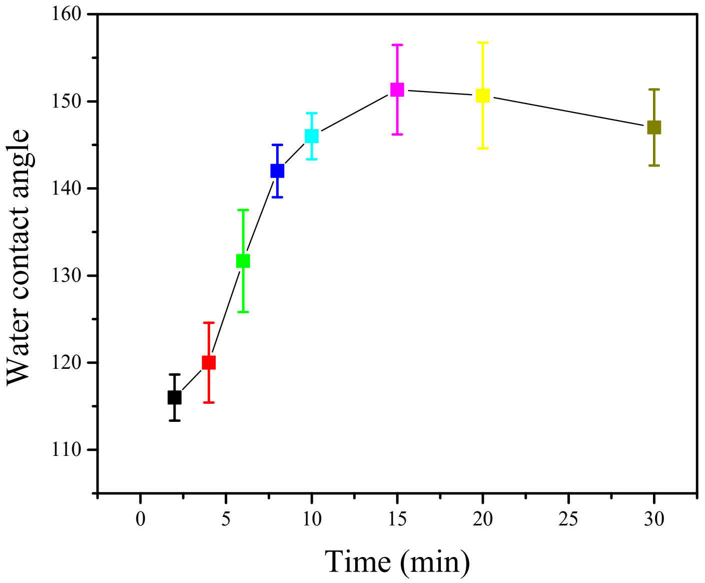

Figure S4. Water contact angle of cellulose paper changing with immersion time from 2 min to 30 min.

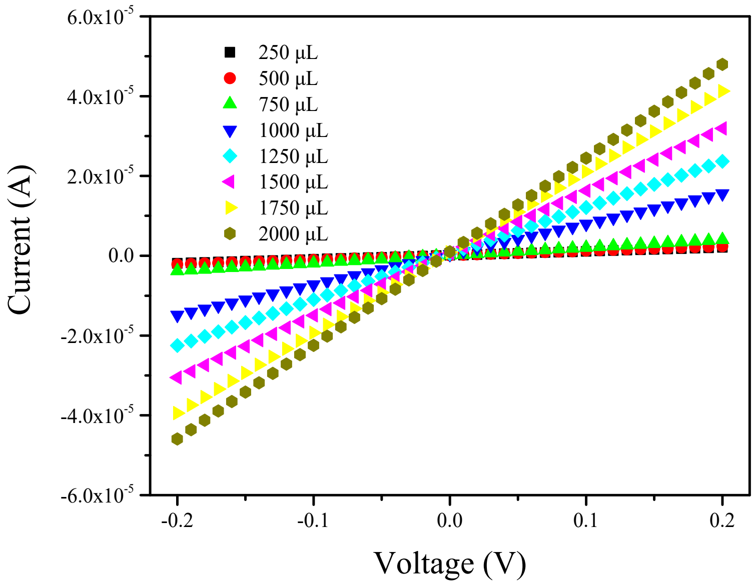

Figure S5. The I_{DS}–V_{DS} of HPFET modified with different volume of SWNTs-PCA.

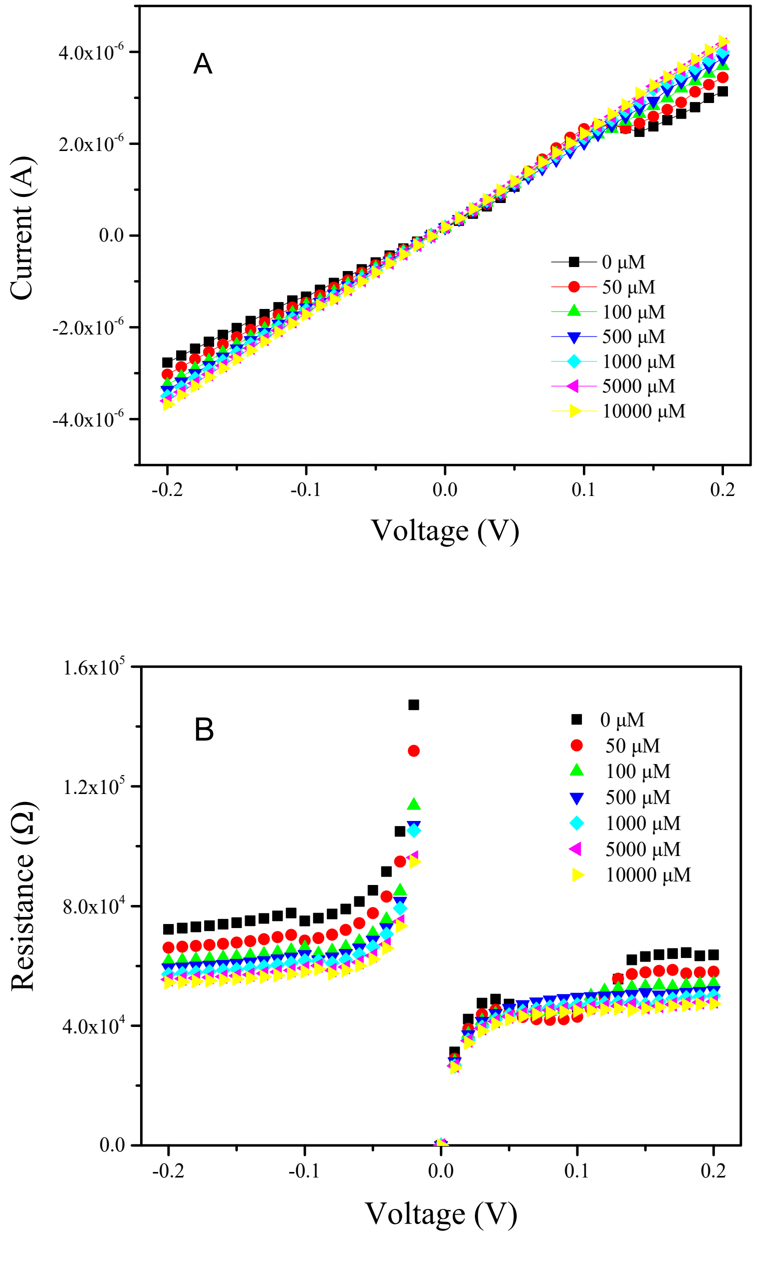
A
B
Current (A)
Voltage (V)
Resistance (Ω)
4.0x10^-6
2.0x10^-6
0.0
-2.0x10^-6
-4.0x10^-6
-0.2
-0.1
0.1
0.2
1.6x10^5
1.2x10^5
8.0x10^4
4.0x10^4
0 μM
50 μM
100 μM
500 μM
1000 μM
5000 μM
10000 μM

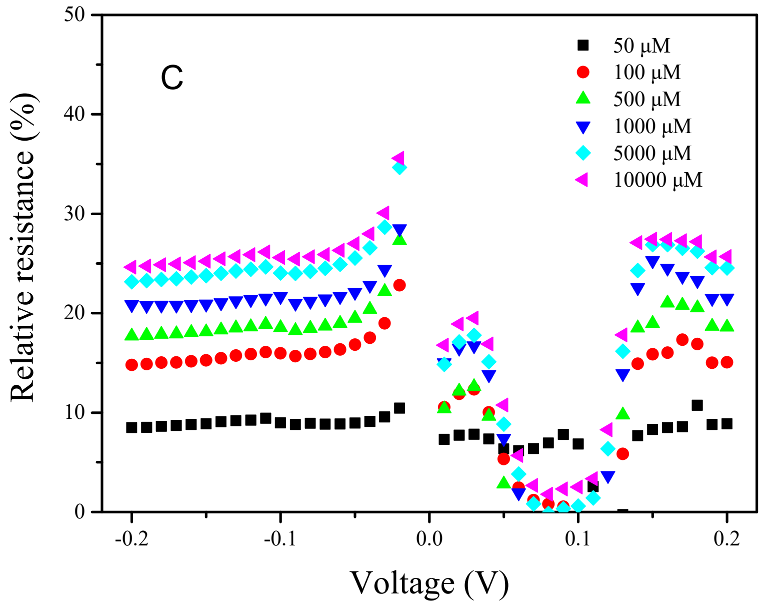

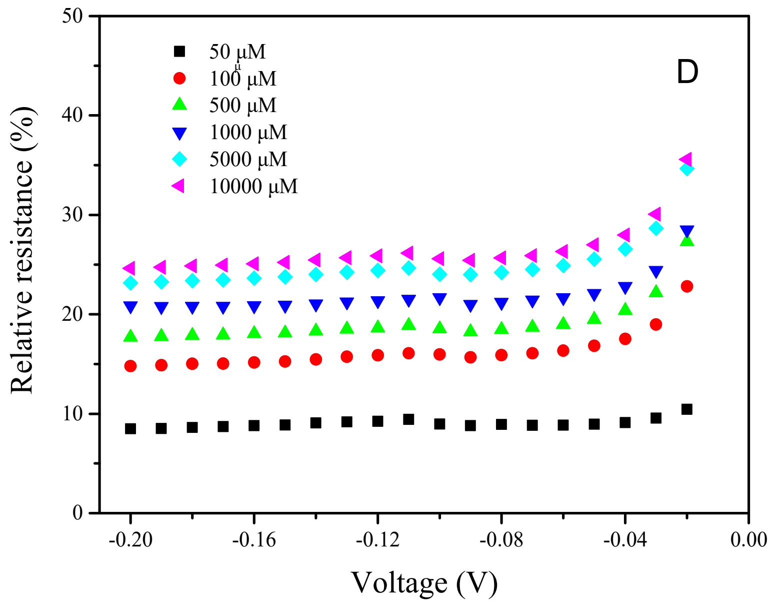

Figure S6. (A)The current-voltage of SPFET/SWNTs-PCA/DNAzyme exposure to different Ca^{2+} concentrations; (B)The resistances at each voltages after SPFET/SWNTs-PCA/DNAzyme exposure to different Ca^{2+} concentrations; (C)The relative resistances

ranging from -0.2 V to 0.2 V after SPFET/SWNTs-PCA/DNAzyme exposure to different Ca^{2+} concentrations. (D) Relative resistances of SPFET/sSWNT-PCA/DNAzyme ranging from -0.2 V to 0 V.

The cleavage efficiency of DNAzyme is associated with Ca^{2+} concentration and incubation time. Three different Ca^{2+} concentrations (100, 1000, and 10000 μM) were measured by SPFET/SWNTs-PCA/DNAzyme with varying incubation times in the range from 0 to 11 min. As shown in Figure S10, the relative resistance of SPFET/SWNTs-PCA/DNAzyme was proportional to Ca^{2+} concentrations and incubation time. For the same Ca^{2+} concentration, the relative resistance increased with incubation time, but the growth rate decreased significantly when the incubation time was higher than 7 min. For this reason, 7 min was chosen as the optimal incubation time for the experiment.

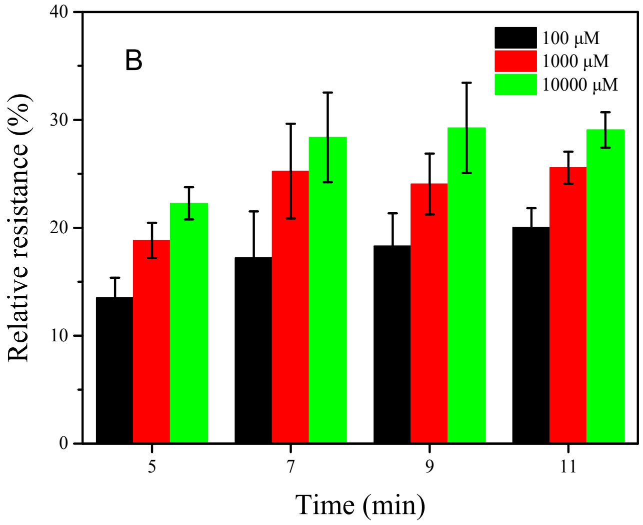

Figure S7. Relative resistances of SPFET/SWNTs-PCA/DNAzyme changing with the

incubation time in the range from 0 to 11 min for three different Ca^{2+} concentrations.

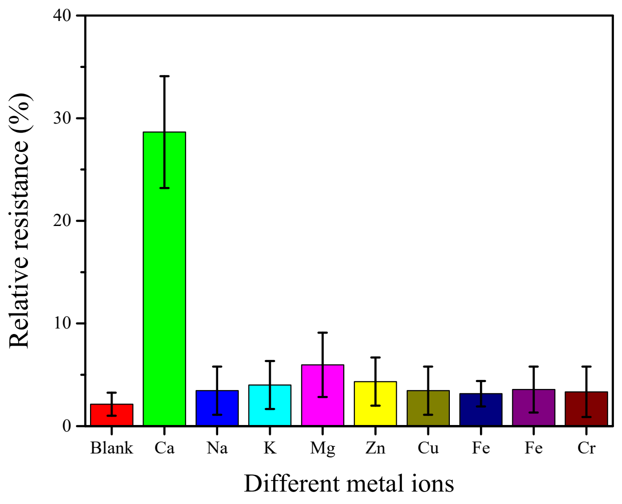

Figure S8. Relative resistances of SPFET/SWNT/DNAzyme before and after incubation in 10 mM solutions of different metal ions including Na^{+}, K^{+}, Mg^{2+}, Zn^{2+}, Cu^{2+}, Fe^{2+}, Fe^{3+}, and Cr^{3+}.

For SPFET/SWNTs-PCA/DNAzyme, the regression equations were $y_1 = 22.132x + 39.265$ and $y_2 = 5.4265x + 23.577$ with the R-squared of 0.9939 and 0.9782, respectively, which the detection limit was 10.7 μM(N/S=3). For SPFET/SWNTs-PCA/Nonzyme, the relative resistance revealed excellent relationship with the logarithm of Ca^{2+} concentration in the range of 0.025 mM to 10 mM. The linear regression equation was $y = 2.3384x + 4.5091$ with the linear regression correlation coefficient of 0.987.

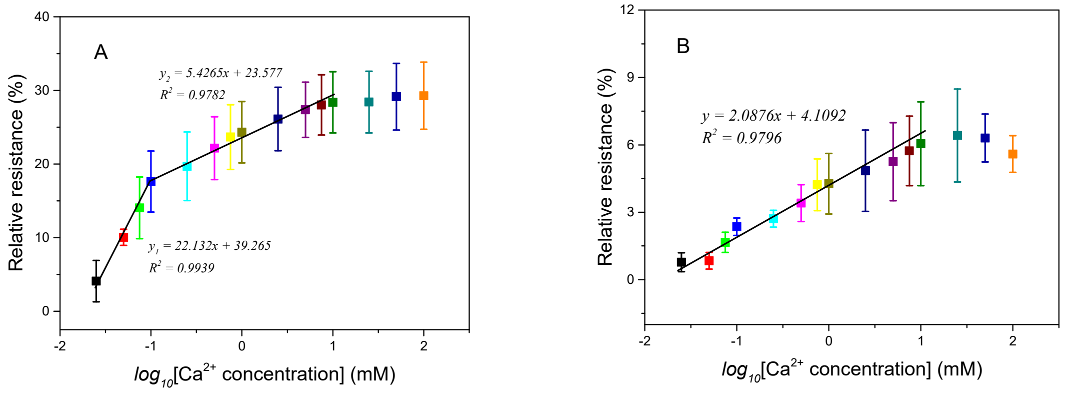

Figure S9. The linear relationship between the relative resistances of SPFET/SWNTs-PCA/DNAzyme(A) and SPFET/SWNTs-PCA/Nonzyme(B) and the logarithm of the Ca^{2+} concentration.

Table S2. Comparison of the performances of Dual-BioFET and other reported sensors for Ca^{2+} determination.

Sensor	Method	pH	Time (min)	Linear range (μM)	LOD (μM)	Ref.
Ca-ISE	Potential	2.0	1.67	3.1×10^{-3}～3.2×10^{4}	-	1
Ca^{2+}-SCISE	Potential	-	1.0	1.0×10^{-1}～1.0×10^{4}	5.00	2
ECL-based ion-selective nano-optodes	Potential	7.4	-	1.0×10^{0}～5.0×10^{2}	0.11	3
Fe_2O_3-ZnO NRs/FET	Curent	7.6	-	1.0×10^{1}～3.0×10^{3}	0.05	4
Co_3O_4 CNT	Curent	-	0.84	1.0×10^{2}～1.1×10^{4}	3.80	5
IS-OECT	Curent	-	20	1.0×10^{1}～1.0×10^{4}		6
CD-EGTA	Fluorescent	7	240	1.5×10^{1}～3.0×10^{2}	0.38	7
DLLME	UV-visiable	12.8	30	1.5×10^{0}～3.7×10^{1}	0.43	8
SD-ISO	Fluorescence	6.0-8.0	5	1.0×10^{1}～1.0×10^{6}	9.30	9
SA-4CO_2Et	Fluorescence	4.0–10.0	240	6.0×10^{2}～3.0×10^{3}	-	10
DNAzyme/SWNT/FET	Curent	6.9	9	1.0×10^{1}～1.0×10^{3}	7.20	11
Dual BioFET	Curent	7.4	7	2.5×10^{1}～5.0×10^{3}	10.70	This work

CD-EGTA: carbon dot-ethylenebis(oxyethylenenitrilo)tetraacetic acid; ISO: ion-selective optode; SD: solvatochromic

dye; ISE: potentiometry based on a calcium-selective electrode; SCISE: solid-contact calcium-selective electrode; IS-OECT: ion-selective organic electrochemical transistor; SA: salicyladazine; ECL: electrochemiluminescence;

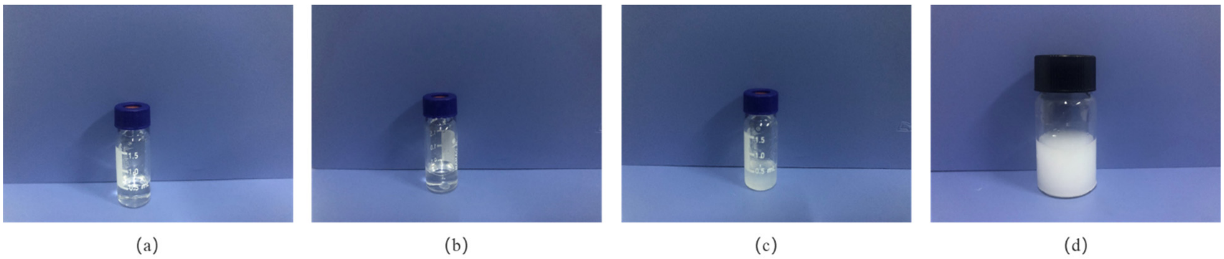

Figure S10. Preparation of superhydrophobic solution: (a) pure OTS; (b) OTS added with water; (c) mixing by vortex and sonication; and (d) incubation for 2 h under ambient conditions and dilution with hexane.

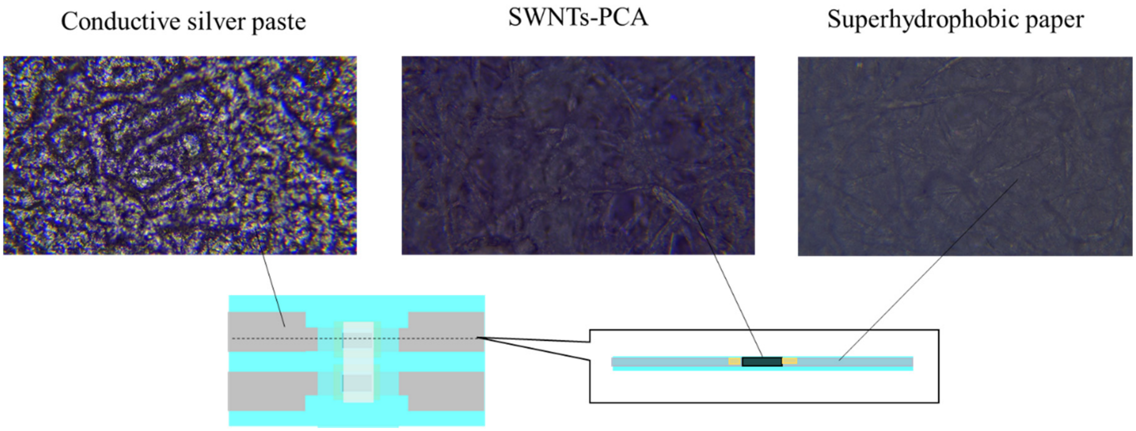

Figure S11. Structural diagram and microscope photographs of SPFET/SWNTs-PCA.

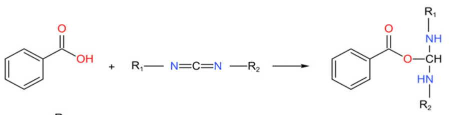 (S1)

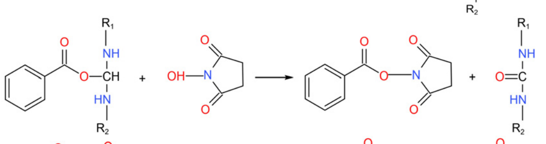 (S2)

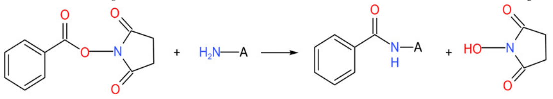 (S3)

References

1. Liu, S.; Ding, J.; Qin, W. Current pulse based ion-selective electrodes for chronopotentiometric determination of calcium in seawater. *Anal. Chim. Acta* **2018**, *1031*, 67–74, https://doi.org/10.1016/j.aca.2018.06.018.
2. Ocaña, C.; Abramova, N.; Bratov, A.; Lindfors, T.; Bobacka, J.J.T. Calcium-selective electrodes based on photo-cured polyurethane-acrylate membranes covalently attached to methacrylate functionalized poly (3, 4-ethylenedioxythiophene) as solid-contact. *Talanta* **2018**, *186*, 279–285.
3. Zhang, J.; Chen, Y.; Fang, D. Electrochemiluminescence in Luminol-based calcium-selective nanoparticles for the determination of calcium ions. *J. Electroanal. Chem.* **2020**, *878*, 114671, https://doi.org/10.1016/j.jelechem.2020.114671.
4. Ahmad, R.; Tripathy, N.; Ahn, M.-S.; Yoo, J.-Y.; Hahn, Y.-B. Preparation of a Highly Conductive Seed Layer for Calcium Sensor Fabrication with Enhanced Sensing Performance. *ACS Sens.* **2018**, *3*, 772–778, https://doi.org/10.1021/acssensors.7b00900.
5. Yuan, H.; Ma, C.; Geng, J.; Zhang, L.; Cui, H.; Liu, C. Preparation of Co_3O_4 conical nanotube and its application in calcium ion biosensor. *Appl. Phys. A* **2018**, *124,* 101, https://doi.org/10.1007/s00339-017-1460-x.
6. Keene, S.; Fogarty, D.; Cooke, R.; Casadevall, C.D.; Salleo, A.; Parlak, O. Wearable Organic Electrochemical Transistor Patch for Multiplexed Sensing of Calcium and Ammonium Ions from Human Perspiration. *Adv. Heal. Mater.* **2019**, *8*, e1901321, https://doi.org/10.1002/adhm.201901321.
7. Yue, J.; Li, L.; Cao, L.; Zan, M.; Yang, D.; Wang, Z.; Chang, Z.; Mei, Q.; Miao, P.; Dong, W.-F. Two-Step Hydrothermal Preparation of Carbon Dots for Calcium Ion Detection. *ACS Appl. Mater. Interfaces* **2019**, *11*, 44566–44572, https://doi.org/10.1021/acsami.9b13737.
8. Peng, B.; Zhou, J.; Xu, J.; Fan, M.; Ma, Y.; Zhou, M.; Li, T.; Zhao, S. A smartphone-based colorimetry after dispersive liquid–liquid microextraction for rapid quantification of calcium in water and food samples. *Microchem. J.* **2019**, 149, https://doi.org/10.1016/j.microc.2019.104072.
9. Shibata, H.; Ikeda, Y.; Hiruta, Y.; Citterio, D. Inkjet-printed pH-independent paper-based calcium sensor with fluorescence signal readout relying on a solvatochromic dye. *Anal. Bioanal. Chem.* **2020**, *412*, 3489–3497.
10. Gao, M.; Li, Y.; Chen, X.; Li, S.; Ren, L.; Tang, B.Z. Aggregation-Induced Emission Probe for Light-Up and in Situ Detection of Calcium Ions at High Concentration. *ACS Appl. Mater. Interfaces* **2018**, *10*, 14410–14417, https://doi.org/10.1021/acsami.8b00952.
11. Wang, H.; Luo, Q.; Zhao, Y.; Nan, X.; Zhang, F.; Wang, Y.; Wang, Y.; Hua, D.; Zheng, S.; Jiang, L.; et al. Electrochemical device based on nonspecific DNAzyme for the high-accuracy determination of Ca2+ with Pb2+ interference. *Bioelectrochemistry* **2020**, *140,* 107732, https://doi.org/10.1016/j.bioelechem.2020.107732.
